# Explicit scheme for solving variable-order time-fractional initial boundary value problems

**DOI:** 10.1038/s41598-024-55943-4

**Published:** 2024-03-05

**Authors:** Asia Kanwal, Salah Boulaaras, Ramsha Shafqat, Bilal Taufeeq, Mati ur Rahman

**Affiliations:** 1 School of Mathematical Sciences, University of Electronic Science and Technology of China, Chengdu, 611731 Sichuan People’s Republic of China; 2 Department of Mathematics, College of Science, Qassim University, 51452 Buraydah, Saudi Arabia; 3 Department of Mathematics and Statistics, The University of Lahore, Sargodha, 40100 Pakistan; 4 Department of Mathematics, Government College University Lahore Pakistan, Lahore, Punjab Pakistan; 5 School of Mathematical Sciences, Jiangsu University, Zhenjiang, 212013 Jiangsu People’s Republic of China; 6 Department of Computer Science and Mathematics, Lebanese American University, Beirut, Lebanon

**Keywords:** Fractional derivatives, Caputo derivative, Explicit scheme, Stability analysis, Initial boundary value problem, Fractional diffusion equations, Engineering, Mathematics and computing, Physics, Engineering, Mathematics and computing, Physics

## Abstract

The creation of an explicit finite difference scheme with the express purpose of resolving initial boundary value issues with linear and semi-linear variable-order temporal fractional properties is presented in this study. The rationale behind the utilization of the Caputo derivative in this scheme stems from its known importance in fractional calculus, an area of study that has attracted significant interest in the mathematical sciences and physics. Because of its special capacity to accurately represent physical memory and inheritance, the Caputo derivative is a relevant and appropriate option for representing the fractional features present in the issues this study attempts to address. Moreover, a detailed Fourier analysis of the explicit finite difference scheme’s stability is shown, demonstrating its conditional stability. Finally, certain numerical example solutions are reviewed and MATLAB-based graphic presentations are made.

## Introduction

Fractional calculus (FC), an extension of classical calculus that involves the integration and differentiation of fractional order, has a rich history dating back to 1695, when the concept of the semi-derivative was first discussed in correspondence between G. W. Leibniz and Marquis de L’Hospital^1^. Since then, notable mathematicians, including Euler, Liouville, Laplace, Riemann, Grunwald, and Letnikov, have contributed to the development of fractional operators^2–5^. The theory of fractional calculus has undergone rapid growth in the 19th century, with applications in various fields, such as fractional geometry, fractional differential equations, and fractional dynamics^6–8^. Today, fractional calculus finds numerous uses in contemporary engineering and research. Methods and tools of fractional calculus are employed in a wide range of fields, including rheology, viscoelasticity, acoustics, optics, chemical and statistical physics, robotics, control theory, electrical and mechanical engineering, and bioengineereing^9–12^. The authors of^13^ considered a 4D memrister system and analyzed its bifurcation, chaos and implement its circuit along with dynamical investigations. The authors of^14^ discussed in their book with the goal of filling the knowledge gap and giving readers a thorough and organized explanation of the key concepts and uses of fractional calculus. A bibliographic review of fractional-order control laws for robotic manipulators, robot vehicles, man-robot systems, and biologically inspired robots was presented by the authors^15^. The authors of^16^ utilized a data-driven approach, the study computes inflation expectations, the monetary policy transparency index, and associated volatility spillover effects. The analysis focuses on the optimization processes of monetary policy transparency that impact inflation and inflation expectations volatility. The most recent advancements and patterns in the use of FC in biology and biomedicine are reviewed by the writers in a paper they published. Nature frequently demonstrates that it operates according to rather basic principles, which cause complex occurrences to arise as a result. Of these, the paper discusses the characteristics of respiratory lung tissue, whose inherent solutions non-integer parametric models and non-integer differ-integral solutions-occur in the middle of FC^17^.

The application of fractional calculus spans various fields and has gained significant attention in recent years. In physics, fractional calculus has been used to describe anomalous diffusion processes, fractional quantum mechanics, and viscoelasticity in materials^18,19^. In mathematical biology, fractional calculus has been employed to model the dynamics of populations, the spread of infectious diseases, and the behavior of biological networks^20,21^. In finance and economics, fractional calculus has been utilized to analyze stock market volatility, option pricing, and fractional order economic models^22,23^. Furthermore, fractional calculus has found applications in image processing, signal processing, control systems, and many other areas. Its ability to capture memory and long-range dependence provides a powerful tool for modeling complex phenomena in diverse disciplines. In the context of fractional calculus, Caputo derivatives are commonly used^24^.

Various numerical methods have been proposed by several authors to solve the fractional diffusion equation. Chen et al.^25^, Birajdar and Dhaigude^26^, Zhang and Liu^27^, Liu et al.^28^, and Lin and Xu^29^ developed explicit finite difference schemes for solving the fractional diffusion equation, while Birajdar^30^ obtained stability for a highly nonlinear time fractional diffusion equation. Furthermore, Dhaigue and Birajdar^31^ applied the discrete Adomian decomposition method to solve various types of fractional partial differential equations. Recently, Kumar et al.^32^ obtained analytical solutions for fractional differential equations. Despite these advances, fractional calculus is still relatively unknown and has only recently gained widespread applications^33^.

Luo et al.^34^ proposed a method for dealing with a specific type of nonlinear fractional difference system that has variable order and fixed initial values. In another study, Luo et al.^35^ investigated a stochastic fractional differential equation (SFDE) with time delays, specifically stochastic Hilfer-type SHFDEs with non-Lipschitz coefficients. Using the Laplace transform and mathematical inequalities, the authors derived an implicit solution for the SHFDEs and presented an averaging principle^36^. Additionally, Luo et al.^37^ proposed the finite-time stability of stochastic fractional-order delay differential equations. Zou et al.^38^ also derived an averaging principle for the system using mathematical inequalities and novel assumptions. Furthermore, Huang et al.^39^ studied another type of SFDEs called conformable fractional stochastic differential equations (CFSDEs) that are driven by fractional Brownian motion with infinite delay. The authors used mathematical methods and a fixed-point theorem to investigate the existence of solutions and the controllability of the system.

The aim of this study is to address two significant challenges in the field of fractional differential equations. Firstly, we tackle the lack of stability analysis in many existing methods for solving such equations. To overcome this limitation, we have developed a scheme specifically tailored for variable-order time fractional initial boundary value problems. Additionally, we have conducted a comprehensive stability analysis of the explicit finite difference scheme. Secondly, our scheme is applicable to both linear and semi-linear equations. However, this paper focuses on presenting numerical results related to the application of our scheme to linear equations, an area that has received less attention compared to similar schemes applied to semi-linear equations. Linear time fractional equations offer several advantages, including easier solvability using standard numerical methods, a simpler mathematical structure, and the ability to model a broader range of physical and biological processes. In contrast, semi-linear time fractional equations are typically limited to more specific applications and present challenges in terms of well-posedness. By addressing these difficulties, our study contributes to the advancement of understanding and application of fractional differential equations. It is important to remember that among the definitions of the fractional derivative, the Caputo definition is the one that is most frequently applied. In terms of mathematics, the Caputo definition is more rigorous than the Riemann-Liouville definition for more details the readers can be found in^40^.

An explicit finite difference strategy for fractional-order equations may have the following possible benefits over alternative approaches to a thorough stability analysis:

Explicit stability criteria designed especially for finite difference schemes used to fractional-order equations are provided by the technique. This provides a precise guideline for assessing stability, making the evaluation process easier than with more generic stability evaluations that do not take into account the special qualities of fractional-order equations. It permits an in-depth numerical stability analysis of the considered finite difference scheme. The approach enables a tailored study with an emphasis on the finite difference scheme’s stability behavior in the setting of fractional-order equations. With this focused approach, the analysis is customized to meet the unique needs and difficulties presented by fractional-order dynamics, which could lead to more insightful and accurate findings. The method can verify if the finite difference scheme is stable by comparing it to theoretical stability bounds or established stability criteria through a thorough stability analysis. For the numerical solution derived from the scheme to be accurate and reliable, this validation phase is essential. It makes it easier to find stable areas in the finite difference scheme’s parameter space, which helps choose the right parameters to guarantee stable numerical solutions. This makes it possible to optimize and tune parameters effectively, which enhances the accuracy and performance of computations. The approach sheds light on possible drawbacks and restrictions in the use of the finite difference strategy for fractional-order equations, as well as its stability bounds. For the numerical solution approach to remain robust and to prevent numerical instabilities, it is imperative to comprehend these stability constraints. The approach makes it possible to optimize scheme parameters to improve stability while preserving computational efficiency, based on the findings of the stability study. To obtain stable and precise numerical solutions, this optimization procedure may include modifying time step sizes, spatial discretization techniques, or other numerical factors. These benefits mean that carrying out a thorough stability analysis of an explicit finite difference scheme for fractional-order equations offers important information and direction for numerical simulations, guaranteeing the accuracy, stability, and dependability of the numerical solution technique in real-world scenarios.

The paper is organized as follows: Section 2 outlines the development of an explicit finite difference scheme, which employs Caputo definition for the time fractional derivative, and the central difference approximation for the space derivative. Additionally, the stability of the scheme is discussed in this section. In Section 3, some examples texted for the validation of the considered scheme. Lastly, we concludes our work in Section 4.

## Methodology

In this section, we discuss the explicit finite difference scheme for solving linear or semi-linear variable-order time fractional differential equations. In the end of this section, we discuss the stability of the proposed scheme.

### Explicit finite difference scheme

Consider the variable-order time fractional semi-linear differential equation

1 $$\begin{aligned} \dfrac{\partial ^{{\gamma }(x,t)} \psi (x,t)}{\partial {t}^{\gamma (x,t)}}=a(x,t)\psi _{xx}+f(\psi ), \end{aligned}$$

where

$$\begin{aligned}{} & {} 0<x<L_x, \quad 0<t\le {T},\quad 0<\gamma (x,t)\le {1}, \\{} & {} \psi (x,0)=\psi (x), \\{} & {} \psi (0,t)=0=\psi (L_x,t), \end{aligned}$$

or

$$\begin{aligned} \psi (0,t)=0=\dfrac{\partial \psi (L_x,t)}{\partial x}. \end{aligned}$$

The function $f(\psi )$ is nonlinear. Without $f(\psi )$ function, the Eq. (1) becomes linear.

### Discretization

Let $[0,L_x]$ be the domain of interest, first, we discretize this domain. To do this, let us define $x_i=ih$, where $0\le {i}\le {M}$, $Mh=L_x$, $t_j=jk$, $0\le {j} \le {N}$, $Nk=T$, where *h* is the space step length and *k* is time step size. Suppose that $\psi _i^j$ be the numerical approximation of $\psi (x_i,t_j)$ and $f_i^j({\psi _i^j})=f(x_i,t_j,\psi _i^j)$. Further, suppose that the non-linear function $f_i^j({\psi _i^j})$ satisfies the Lipschitz condition. $|f_i^j(\psi _i^j)-f_i^j(\bar{\psi }_i^j)|\le {L_p}|\psi _i^j-\bar{\psi }_i^j|, \ L_p$ is a non-negative Lipschitz constant.

### Development of the scheme

Considering Eq. (1) in which $\gamma (x,t)$ represents the fractional order. The fractional derivative of order $\gamma {(x,t)}$ is defined by Coimbra in terms of Caputo and is defined as

2 $$\begin{aligned} \dfrac{\partial ^\gamma {\psi (x,t)}}{\partial {t^\gamma }}:={\left\{ \begin{array}{ll} \dfrac{1}{\Gamma (1-\gamma (x,t))}\displaystyle \int _0^t \dfrac{\psi _\xi d\xi }{(t-\xi )^{\gamma (x,t)}} \quad &{}\text {if} \quad 0<\gamma (x,t)<1, \\ \psi _t \quad &{}\text {if} \quad \gamma (x,t)=1.\\ \end{array}\right. } \end{aligned}$$

Transforming (1) using (2)

$$\begin{aligned} \dfrac{\partial ^{\gamma (x_i,t_{j})}\psi (x_i,t_{j})}{\partial t^{\gamma (x_i,t_{j})}}&=\dfrac{1}{\Gamma (1-\gamma (x_i,t_{j}))}\int _0^{t_j} \dfrac{\psi _\xi d\xi }{(t_{j}-\xi )^{\gamma (x_i,t_{j})}},\\&=\dfrac{1}{\Gamma (1-\gamma (x_i,t_{j}))}\sum _{l=0}^{j-1}\int _{(l)k}^{(l+1)k} \dfrac{\partial \psi (x_i,\xi )}{\partial \xi }\times \dfrac{d\xi }{(t_{j}-\xi )^{\gamma (x_i,t_{j})}}.\\ \end{aligned}$$

Using forward difference formula for temporal derivative

$$\begin{aligned}&=\dfrac{1}{\Gamma (1-\gamma (x_i,t_{j}))}\sum _{l=0}^{j-1}\dfrac{\psi _i^{l+1}-\psi _i^l}{k}\int _{(l)k}^{(l+1)k} \dfrac{d\xi }{(t_{j}-\xi )^{\gamma (x_i,t_{j})}},\\&=\dfrac{1}{\Gamma (1-\gamma (x_i,t_{j}))}\sum _{l=0}^{j-1}\dfrac{\psi _i^{l+1}-\psi _i^l}{k}\int _{(j-l-1)k}^{(j-l)k} \dfrac{d \eta }{ \eta ^{\gamma (x_i,t_j)}}. \end{aligned}$$

Equivalently, the above expression can also be written as

$$\begin{aligned} \dfrac{\partial ^{\gamma (x_i,t_{j})}\psi (x_i,t_{j})}{\partial t^{\gamma (x_i,t_{j})}}&=\dfrac{1}{\Gamma (1-\gamma (x_i,t_{j}))}\sum _{l=0}^{j-1}\dfrac{\psi _i^{j-l}-\psi _i^{j-l-1}}{k}\int _{(l)k}^{(l+1)k} \eta ^{-\gamma (x_i,t_j)}d\eta . \end{aligned}$$

Upon Integrating the above equation, we obtain

$$\begin{aligned} \dfrac{\partial ^{\gamma (x_i,t_{j})}\psi (x_i,t_{j})}{\partial t^{\gamma (x_i,t_{j})}}&=\dfrac{1}{\Gamma (1-\gamma (x_i,t_{j}))}\sum _{l=0}^{j-1}\dfrac{\psi _i^{j-l}-\psi _i^{j-l-1}}{k}\\ & \quad \times \dfrac{((l+1)k)^{1-\gamma (x_i,t_j)}-((l)k)^{{1-\gamma (x_i,t_j)}}}{{1-\gamma (x_i,t_j)}}. \end{aligned}$$

Using $\Gamma {(1+\gamma )}=\gamma \Gamma {(\gamma )}$ and expanding the summation for $l=0$, we reach at

$$\begin{aligned} \dfrac{\partial ^{\gamma (x_i,t_{j})}\psi (x_i,t_{j})}{\partial t^{\gamma (x_i,t_{j})}}&=\dfrac{1}{\Gamma (2-\gamma (x_i,t_{j}))}\dfrac{\psi _i^{j}-\psi _i^{j-1}}{k}\quad {k^{1-\gamma (x_i,t_j)}}\\ & \quad + \dfrac{1}{\Gamma (2-\gamma (x_i,t_{j}))}\sum _{l=1}^{j-1}\dfrac{\psi _i^{j-l}-\psi _i^{j-l-1}}{k}\\ & \quad \times (((l+1)k)^{1-\gamma (x_i,t_j)}-((l)k)^{{1-\gamma (x_i,t_j)}}),\\ & =\dfrac{{k^{-\gamma (x_i,t_j)}}}{\Gamma (2-\gamma (x_i,t_{j}))}[(\psi _i^j-\psi _i^{j-1})\\ & \quad +\sum _{l=1}^{j-1}(\psi _l^{j-l}-\psi _l^{j-l-1})\times (((l+1)k)^{1-\gamma (x_i,t_j)}-((l)k)^{{1-\gamma (x_i,t_j)}})], \end{aligned}$$

Replace *j* by $$j+1$$

$$\begin{aligned} ,\dfrac{\partial ^{\gamma (x_i,t_{j+1})}\psi (x_i,t_{j+1})}{\partial t^{\gamma (x_i,t_{j+1})}}&=\dfrac{{k^{-\gamma (x_i,t_{j+1})}}}{\Gamma (2-\gamma (x_i,t_{j+1}))}[(\psi _i^{j+1}-\psi _i^{j}) \\ & \quad +\sum _{l=1}^{j}(\psi _l^{j+1-l}-\psi _l^{j-l})\times (((l+1)k)^{1-\gamma (x_i,t_{j+1})}-((l)k)^{{1-\gamma (x_i,t_{j+1})}})], \end{aligned}$$

or

3 $$\begin{aligned} \dfrac{\partial ^{\gamma (x_i,t_{j+1})}\psi (x_i,t_{j+1})}{\partial t^{\gamma (x_i,t_{j+1})}}=\dfrac{{k^{-\gamma (x_i,t_{j+1})}}}{\Gamma (2-\gamma (x_i,t_{j+1}))}[(\psi _i^{j+1}-\psi _i^{j})+ \sum _{l=1}^{j}(\psi _l^{j+1-l}-\psi _l^{j-l})b_l^{i,{j+1}}], \end{aligned}$$

where

$$\begin{aligned} b_l^{i,{j+1}}=((l+1)k)^{1-\gamma (x_i,t_{j+1})}-((l)k)^{1-\gamma (x_i,t_{j+1})},\quad i=0,1,\ldots ,M,\quad j=0,1,\ldots ,N. \end{aligned}$$

Discretization of non-linear function $f(\psi )$ is given as

4 $$\begin{aligned}{} & {} f(x_i,t_j,\psi (x_i,t_j))=f_i^j(\psi _i^j)+O(k), \nonumber \\{} & {} \psi _{xx}=\dfrac{\psi _{i-1}^j-2\psi _i^j+\psi _{i+1}^j}{h^2}+O(h^2). \end{aligned}$$

Using Eqs. (3) and (4), Eq. (1) takes the form

$$\begin{aligned} \dfrac{k^{-\gamma _i^{j+1}}}{\Gamma (2-\gamma _i^{j+1})}\Big [(\psi _i^{j+1}-\psi _i^{j}) +\sum _{l=1}^{j}(\psi _l^{j+1-l}-\psi _l^{j-l})b_l^{i,{j+1}}\Big ]&=a_i^j\left( \dfrac{\psi _{i-1}^j-2\psi _i^j +\psi _{i+1}^j}{h^2}\right) +f_i^j(\psi _i^j), \end{aligned}$$

or

$$\begin{aligned} \psi _i^{j+1}-\psi _i^{j}+\sum _{l=1}^{j}(\psi _l^{j+1-l}-\psi _l^{j-l})b_l^{i,{j+1}}&=r_i^{j+1}\left( \psi _{i-1}^j-2\psi _i^j+\psi _{i+1}^j\right) +k^{\gamma _i^{j+1}}\Gamma (2-\gamma _i^{j+1})f_i^j(\psi _i^j), \end{aligned}$$

where

$$\begin{aligned} r_i^{j+1}=\dfrac{a_i^jk^{\gamma _i^{j+1}}\Gamma (2-\gamma _i^{j+1})}{h^2}. \end{aligned}$$

Re-arranging the terms, Eq. (1) can be written as

5 $$\begin{aligned} \psi _i^{j+1}= & {} r_i^{j+1}\psi _{i-1}^j+(1-2r_i^{j+1})\psi _i^j+r_i^{j+1}\psi _{i+1}^j -\sum _{l=1}^{j}[\psi _l^{j+1-l}-\psi _l^{j-l}]b_l^{i,{j+1}}+f_i^j(\psi _i^j)k^{\gamma _i^{j+1}}\Gamma (2-\gamma _i^{j+1}), \end{aligned}$$

6 $$\begin{aligned} \psi _i^0= & {} h(x_i) \quad i=0,1,\ldots ,M, \end{aligned}$$

7 $$\begin{aligned} \psi _0^j= & {} 0=\psi _M^j \quad j=0,1,\ldots ,N. \end{aligned}$$

The comparison of the proposed method with previous techniques is given in the Table 1 below.

**Table 1:** Proposed method comparison with the previous methods.

Strategies	Methodology	Benefits and shortcomings
Alia et al.^41^ proposed a new group of iterative techniques to address the numerical solution of a two-dimensional sub-diffusion equation that involves fractional derivatives and specific boundary conditions	These iterative schemes are designed to provide a robust and efficient means of solving two-dimensional sub-diffusion equations	This method is computationally efficient, but no stability analysis has been performed
Oderinu et al.^42^ proposed a method that focuses on finding approximate solutions to linear time fractional differential equations under specific boundary conditions	It investigates a numerical approach for the solution of linear time fractional differential equations of the Caputo type. The results of the research culminated in the establishment of a theorem that showcases the Kamal transform of the nth-order Caputo derivatives	The proposed numerical scheme provides highly accurate solutions for linear time fractional differential equations. However, no stability analysis of the scheme has been performed. This method is limited in scope and can only be applied to linear time fractional differential equations
Proposed	The proposed method uses the central finite difference method for approximating the second-order spatial derivative and forward difference for approximating the Caputo derivative of time. This combination of techniques allows for an efficient and accurate numerical approximation of the solutions to linear/semi-linear time fractional differential equations	This numerical scheme is versatile and can be applied to both linear and semi-linear equations, providing a flexible solution for a range of problems. The stability of the method has been rigorously verified, ensuring reliable results for a wide range of parameters. Additionally, the method is not limited by specific boundary conditions, making it suitable for a wide range of applications

The following section evaluates the stability of the discrete equation scheme (5, 6, 7).

### Stability analysis

The most common technique for stability analysis of the explicit finite difference scheme is the Von Neumann stability analysis, which involves linearizing the problem around a steady state and studying the eigenvalues of the resulting linear system^43^. It is also possible to use other stability analysis techniques such as Fourier analysis^44^, Lax-Richtmyer^45^, and Von Neumann Courant-Friedrichs-Lewy (CFL) conditions^46^.

To evaluate the stability of the scheme, we consider $\rho _i^j=\psi _i^j-\varphi _i^j$, where $\varphi _i^j$ is the accurate solution at $(x_i,t_j)$ and apply the Fourier method. The discrete function $\rho ^j(x^*_i)$ is formulated as:

8 $$\begin{aligned} \rho ^j(x^*_i)={\left\{ \begin{array}{ll} \rho _i^j \quad &{}\text {if} \quad x_i-\dfrac{h}{2}<x_i^*\le x_i+\dfrac{h}{2}, \\ 0, \quad &{}\text {if} \quad 0\le x\le \dfrac{h}{2}\quad or\quad L_x-\dfrac{h}{2}<x_i^*\le L_x. \end{array}\right. } \end{aligned}$$

In Fourier series, the function (8) can be expanded

$$\begin{aligned} \rho ^j(x_i^*)=\sum _{m=-\infty }^{\infty }\xi _j(m)e^{\dfrac{2\pi \iota m}{L_x}}, \end{aligned}$$

where

9 $$\begin{aligned} \xi _j(m)=\dfrac{1}{L_x}\int _0^{L_x}\rho ^j(x_i^*)e^{\dfrac{2\pi \iota m}{L_x}}dx,\qquad \Vert \rho ^j(m)\Vert _2^2=\sum _{-\infty }^\infty |\xi _j(m)|^2 . \end{aligned}$$

Properties of the coefficient $r_i^j$ and $d_l^{i,j}$

1. $$r_i^j>0,\quad 0<b_l^{i,j}<d_{l-1}^{i,j}<1,$$
  where $$d_l^{i,{j+1}}=b_l^{i,{j+1}}-b_l^{i,{j+1}},\quad \forall i=1,2,\ldots ,M, l=1,2,\ldots ,N.$$
2. $$0<d_l^{i,j}<1,\quad \sum _{j=0}^{k-1}d_{l+1}^{i,{j+1}}=1-b_l^{i,{j+1}}.$$
  It is easy to prove property (2).

### Stability of the scheme

The stability of the scheme is analyzed in this section. By inserting $\rho _i^j=u_i^j-U_i^j$ into Eqs. (5, 6)

$$\begin{aligned} \rho _i^{j+1}&=r_i^{j+1}\rho _{i-1}^j+(1-2r_i^{j+1})\rho _i^j+r_i^{j+1}\rho _{i+1}^j -\sum _{l=1}^{j}[\rho _i^{j+1-l}-\rho _i^{j-l}]b_l^{i,{j+1}}\\& \quad +k^{\gamma _i^{j+1}}\Gamma (2-\gamma _i^{j+1})\times [f(x_i,t_j,\psi (x_i,t_j)-f_i^j(\psi _i^j)]. \end{aligned}$$

Evaluating sum for $l=j$, we get

$$\begin{aligned} \rho _i^{j+1}&=r_i^{j+1}\rho _{i-1}^j+(1-2r_i^{j+1})\rho _i^j+r_i^{j+1}\rho _{i+1}^j -\sum _{l=1}^{j-1}[\rho _i^{j+1-l}-\rho _i^{j-l}]b_l^{i,{j+1}}-(\rho _i^1-\rho _i^0)b_j^{i,{j+1}}\\ & \quad +k^{\gamma _i^{j+1}}\Gamma (2-\gamma _i^{j+1})\times [f(x_i,t_j,\psi (x_i,t_j)-f_i^j(\psi _i^j)]. \end{aligned}$$

Simplification yields us

10 $$\begin{aligned} \rho _i^{j+1}&=r_i^{j+1}\rho _{i-1}^j+(1-2r_i^{j+1})\rho _i^j+r_i^{j+1}\rho _{i+1}^j-\sum _{l=1}^{j-1}\rho _i^{j+1-l}b_l^{i,{j+1}} -\sum _{l=1}^{j-1}\rho _i^{j-l}b_l^{i,{j+1}}-\rho _i^1b_j^{i,{j+1}}\nonumber \\ & \quad +\rho _i^0b_j^{i,{j+1}}+k^{\gamma _i^{j+1}}\Gamma (2-\gamma _i^{j+1})[f(x_i,t_j,\psi (x_i,t_j)-f_i^j(\psi _i^j)]. \end{aligned}$$

Since

11 $$\begin{aligned} -\sum _{l=1}^{j-1}\rho _i^{j+1-l}b_l^{i,{j+1}}-\rho _i^1b_j^{i,{j+1}}&=-\sum _{l=1}^j\rho _i^{j+1-l}b_l^{i,{j+1}}\nonumber , \\ &=-\sum _{l=0}^{j-1}\rho _i^{j-l}b_{l+1}^{i,{j+1}}\nonumber ,\\ & =-b_1^{i,{j+1}}\rho _i^j-\sum _{l=1}^{j-1}\rho _i^{j-l}b_{l+1}^{i,{j+1}}. \end{aligned}$$

Substituting (11) in Eq. (10), we get

12 $$\begin{aligned} \rho _i^{j+1}&=r_i^{j+1}\rho _{i-1}^j+(1-2r_i^{j+1})\rho _i^j+r_i^{j+1}\rho _{i+1}^j-b_1^{i,{j+1}}\rho _i^j -\sum _{l=1}^{j-1}\rho _i^{j-l}b_{l+1}^{i,{j+1}}+\sum _{l=1}^{j-1}\rho _i^{j-l}b_l^{i,{j+1}}\nonumber \\ & \quad +\rho _i^0b_j^{i,{j+1}}+k^{\gamma _i^{j+1}}\Gamma (2-\gamma _i^{j+1})\times [f(x_i,t_j,\psi (x_i,t_j)-f_i^j(\psi _i^j)]. \end{aligned}$$

Further simplified to obtain

$$\begin{aligned} \rho _i^{j+1}&=r_i^{j+1}\rho _{i-1}^j+(1-b_1^{i,{j+1}}-2r_i^{j+1})\rho _i^j+r_i^{j+1}\rho _{i+1}^j +\sum _{l=1}^{j-1}\rho _i^{j-l}d_{l+1}^{i,{j+1}}+\rho _i^0b_j^{i,{j+1}}\\ & \quad +k^{\gamma _i^{j+1}}\Gamma (2-\gamma _i^{j+1})\times [f(x_i,t_j,\psi (x_i,t_j)-f_i^j(\psi _i^j)], \end{aligned}$$

where

$$\begin{aligned} d_{l+1}^{i,{j+1}}=b_l^{i,{j+1}}-b_{l+1}^{i,{j+1}}. \end{aligned}$$

Let solution at grid points be of the form

13 $$\begin{aligned} \rho _i^j=\xi ^je^{\iota \lambda ih}. \end{aligned}$$

Substituting (13) in Eq. (12)

$$\begin{aligned} \xi ^{j+1}e^{\iota \lambda (ih)}&=r_i^{j+1}\xi ^je^{\iota \lambda (i-1)h}+(1-b_1^{i,{j+1}}-2r_i^{j+1})\xi ^je^{\iota \lambda ih}+r_i^{j+1}\xi ^je^{\iota \lambda (i+1)h}\\ & \quad +\sum _{l=1}^{j-1}\xi ^{j-l}e^{\iota \lambda (ih)}d_{l+1}^{i,{j+1}}+\xi ^0e^{\iota \lambda (ih)}b_j^{i,{j+1}}+ k^{\gamma _i^{j+1}}\Gamma (2-\gamma _i^{j+1})[f(x_i,t_j,\psi (x_i,t_j)-f_i^j(\psi _i^j)]. \end{aligned}$$

Simplifying and re-arranging the terms

$$\begin{aligned} \xi ^{j+1}&=r_i^{j+1}\xi ^j[e^{-\iota \lambda (h)}+e^{\iota \lambda h}]+(1-b_1^{i,{j+1}}-2r_i^{j+1})\xi ^j+\sum _{l=1}^{j-1}\xi ^{j-l}d_{l+1}^{i,{j+1}}+\xi ^0b_j^{i,{j+1}}\\ & \quad + k^{\gamma _i^{j+1}}\Gamma (2-\gamma _i^{j+1})[f(x_i,t_j,\psi (x_i,t_j)-f_i^j(\psi _i^j)]e^{-\iota \lambda h}. \end{aligned}$$

Using identity $e^{ix}=cosx+isinx$, and re-arranging the terms

$$\begin{aligned} \xi ^{j+1}&=2r_i^{j+1}\xi ^j\cos (\lambda h)+(1-b_1^{i,{j+1}}-2r_i^{j+1})\xi ^j+\sum _{l=1}^{j-1}\xi ^{j-l}d_{l+1}^{i,{j+1}}+\xi ^0b_j^{i,{j+1}}\\ & \quad +k^{\gamma _i^{j+1}}\Gamma (2-\gamma _i^{j+1})[f(x_i,t_j,\psi (x_i,t_j)-f_i^j(\psi _i^j)]e^{-\iota \lambda h}. \end{aligned}$$

or

14 $$\begin{aligned} \xi ^{j+1}&=(1-b_1^{i,{j+1}}-4r_i^{j+1}\sin ^2(\dfrac{\lambda h}{2}))\xi ^j+\sum _{l=1}^{j-1}\xi ^{j-l}d_{l+1}^{i,{j+1}}+\xi ^0b_j^{i,{j+1}} +k^{\gamma _i^{j+1}}\Gamma (2-\gamma _i^{j+1})\nonumber \\ & \quad \times [f(x_i,t_j,\psi (x_i,t_j)-f_i^j(\psi _i^j)]e^{-\iota \lambda h} . \end{aligned}$$

The following lemma provides a framework for evaluating the stability of the scheme.

#### **Lemma**

Suppose that $\xi ^j$ be the solution of (14) and $\forall (i,j), r_i^j\le \dfrac{1}{2\sin ^2\Big (\dfrac{\lambda h}{2}\Big )}$ then $|\xi ^j|\le C^*|\xi ^0|,$holds for $j=1,2,.....,N-1.$

#### *Proof*

Using mathematical induction.

Let $$j=0$$, (14) becomes

$$\begin{aligned} \xi ^{1}&=\Big (1-b_1^{i,{1}}-4r_i^{1}\sin ^2\Big (\dfrac{\lambda h}{2}\Big )\Big )\xi ^0+\xi ^0b_0^{i,{1}}+ k^{\gamma _i^{1}}\Gamma (2-\gamma _i^{1})\times [f(x_i,t_0,\psi (x_i,t_0)-f_i^0(\psi _i^0)]e^{-\iota \lambda (h)}, \end{aligned}$$

or

$$\begin{aligned} \xi ^{1}&=\Big (1-b_1^{i,{1}}+b_0^{i,{1}}-4r_i^{1}\sin ^2\Big (\dfrac{\lambda h}{2}\Big )\Big )\xi ^0+ k^{\gamma _i^{1}}\Gamma (2-\gamma _i^{1})\times [f(x_i,t_0,\psi (x_i,t_0)-f_i^0(\psi _i^0)]e^{-\iota \lambda (h)}, \end{aligned}$$

where we have used the result $d_l^{i,{j+1}}=b_{l-1}^{i,{j+1}}-b_{l}^{i,{j+1}}$.

Taking modulus on both sides

$$\begin{aligned} \Big |\xi ^{1}\Big |&=\Big |(1+d_1^{i,1}-4r_i^{1}\sin ^2\Big (\dfrac{\lambda h}{2}\Big ))\xi ^0+ k^{\gamma _i^{1}}\Gamma (2-\gamma _i^{1})\times [f(x_i,t_0,\psi (x_i,t_0)-f_i^0(\psi _i^0)]e^{-\iota \lambda (h)}\Big |,\\ \Big |\xi ^{1}\Big |&\le \Big |(1-4r_i^{1}\sin ^2\Big (\dfrac{\lambda h}{2}\Big ))\Big |\xi ^0+ k^{\gamma _i^{1}}\Gamma (2-\gamma _i^{1})\times \Big |[f(x_i,t_0,\psi (x_i,t_0)-f_i^0(\psi _i^0)]e^{-\iota \lambda h}\Big |,\\ \Big |\xi ^{1}\Big |&\le [2+L_pk^{\gamma _i^{1}}\Gamma (2-\gamma _i^{1})]\Big |\xi ^0\Big |,\\ \Big |\xi ^{1}\Big |&\le C^0\Big |\xi ^0\Big |. \end{aligned}$$

where

$$\begin{aligned} C^0=[2+L_pk^{\gamma _i^{1}}\Gamma (2-\gamma _i^{1})]. \end{aligned}$$

For $j>0$, the Eq. (14) can be written as

15 $$\begin{aligned} \xi ^{j+1}&=(1-b_1^{i,{j+1}}-4r_i^{j+1}\sin ^2(\dfrac{\lambda h}{2}))\xi ^j+\sum _{l=1}^{j-1}\xi ^{j-l}d_{l+1}^{i,{j+1}}+\xi ^0b_j^{i,{j+1}} +k^{\gamma _i^{j+1}}\Gamma (2-\gamma _i^{j+1})\nonumber \\ & \quad \times [f(x_i,t_j,\psi (x_i,t_j)-f_i^j(\psi _i^j)]e^{-\iota \lambda h}. \end{aligned}$$

Let us now assume that the given result holds for *j* and prove it for $j+1$, i.e., it holds $|\xi ^j| \le C|\xi ^0|$ and we are to show that $|\xi ^{j+1}|\le C^*|\xi ^{0}|$. Taking modulus on both sides of (15), i.e.,

$$\begin{aligned} \Big |\xi ^{j+1}\Big |&=\Big |\Big (1-b_1^{i,{j+1}}-4r_i^{j+1}\sin ^2\Big (\dfrac{\lambda h}{2}\Big )\Big )\xi ^j+\sum _{l=1}^{j-1}\xi ^{j-l}d_{l+1}^{i,{j+1}}+\xi ^0b_j^{i,{j+1}}\\ & \quad +k^{\gamma _i^{j+1}}\Gamma (2-\gamma _i^{j+1})\times [f(x_i,t_j,\psi (x_i,t_j)-f_i^j(\psi _i^j)]e^{-\iota \lambda (h)}\Big |,\\ \Big |\xi ^{j+1}\Big |&\le \Big |(1-b_1^{i,{j+1}}-4r_i^{j+1}\sin ^2\Big (\dfrac{\lambda h}{2}\Big )\Big )\xi ^j\Big |+\Big |\sum _{l=1}^{j-1}\xi ^{j-l}d_{l+1}^{i,{j+1}}+\xi ^0b_j^{i,{j+1}}\\ & \quad +k^{\gamma _i^{j+1}}\Gamma (2-\gamma _i^{j+1})\times [f(x_i,t_j,\psi (x_i,t_j)-f_i^j(\psi _i^j)]e^{-\iota \lambda (h)}\Big |.\\ \end{aligned}$$

We know that, $|\xi ^j|\le C^*|\xi ^0|$ for all $k>1$.

$$\begin{aligned} |\xi ^{j+1}|\le |(d_1^{i,{j+1}}-4r_i^{j+1}\sin ^2(\dfrac{\lambda h}{2}))C^*\xi ^0|+\sum _{l=1}^{j-1}d_{l+1}^{i,{j+1}}\bar{C}^*|\xi ^0|+b_l^{i,{j+1}}|\xi ^0|+k^{\gamma _i^{j+1}}\Gamma (2-\gamma _i^{j+1})L_P|\xi ^0|. \end{aligned}$$

Since $$\sum _{l=1}^{j-1}d_{l+1}^{i,{j+1}}=1-b_l^{i,{j+1}}<1$$, because $$0<b_l^{i,{j+1}}<1.$$

$$\begin{aligned} |\xi ^{j+1}|&\le |(2-4r_i^{j+1}\sin ^2(\dfrac{\lambda h}{2}))|C^*_1|\xi ^0|+k^{\gamma _i^{j+1}}\Gamma (2-\gamma _i^{j+1})L_P|\xi ^0|,\\ |\xi ^{j+1}|&\le (\bar{C}_1^*+k^{\gamma _i^{j+1}}\Gamma (2-\gamma _i^{j+1})L_P)|\xi ^0|=C^*|\xi ^o|, \end{aligned}$$

where

$$\begin{aligned} C^* = \bar{C}_1^*+k^{\gamma _i^{j+1}}\Gamma (2-\gamma _i^{j+1})L_P. \end{aligned}$$

The proof follows by induction. $\square$

#### **Theorem**

Explicit finite difference schemes (5,6) to (7) is stable under these circumstances, $r_i^j\le \dfrac{1}{2\sin ^2\big ({\lambda h}/{2}\big )},\quad \forall (i,j)$ $i=1,2,\ldots ,M$; $j=0,1,\ldots ,N$.

#### *Proof*

To prove that the explicit finite difference schemes given in Eqs. (5, 6) to (7) are stable under the condition $r_i^j \le \frac{1}{2\sin ^2\left( \frac{\lambda h}{2}\right) }$ for all (*i*, *j*), where $i=1,2,\dots ,M$ and $j=0,1,\dots ,N$, we can proceed as follows:

Using Eq. (10) and the given lemma, we have:

$$\begin{aligned} |\rho ^j|_2 \le C^|\rho ^0|_2 \quad \text {for } j=1,2,\dots ,N, \end{aligned}$$

To establish stability, we need to show that $|\rho ^j|_2$ remains bounded for all $j=1,2,\dots ,N$, given the stability condition on the coefficients $r_i^j$.

First, let’s define $E^j = |\rho ^j|_2$ for convenience. Our goal is to show that $E^j$ is bounded for all *j*.

Using Eq. (7) and the definition of $|\cdot |2$ norm, we can rewrite $E^j$ as:

$$\begin{aligned} E^j = \left( \sum _{i=1}^M (r_i^j)^2\right) ^{1/2}. \end{aligned}$$

Now, we can analyze the behavior of $E^j$ in terms of $E^{j-1}$. By substituting the expression for $E^j$ and $E^{j-1}$ into equation (5,6), we have:

$$\begin{aligned} E^j=\left( \sum _{i=1}^M \left( \frac{\rho _i^j - \rho _i^{j-1}}{k}\right) ^2\right) ^{1/2}=\frac{1}{k} \left( \sum _{i=1}^M (\rho _i^j - \rho _i^{j-1})^2\right) ^{1/2}=\frac{1}{k} |\rho ^j - \rho ^{j-1}|_2. \end{aligned}$$

Since $|\cdot |_2$ norm is a valid norm, we have the triangle inequality:

$$\begin{aligned} |\rho ^j - \rho ^{j-1}|_2 \le |\rho ^j|_2 + |\rho ^{j-1}|_2. \end{aligned}$$

Therefore, we can bound $E^j$ as follows:

$$\begin{aligned} E^j \le \frac{1}{k} \left( |\rho ^j|_2 + |\rho ^{j-1}|_2\right) \le \frac{1}{k} (C^|\rho ^0|_2 + C^|\rho ^0|_2) \ = \frac{2C}{k}|\rho ^0|_2. \end{aligned}$$

Thus, we have shown that $E^j$ is bounded for all $j=1,2,\dots ,N$.

Therefore, the explicit finite difference schemes (5,6) to (7) are stable under the given condition. This proves the stability of the scheme. $\square$

## Numerical experiments

In this section, we present a numerical solution for variable-order time fractional linear initial boundary value problems using an explicit finite difference scheme. We investigate the influence of the fractional order $\gamma$ by solving problems for different values of $\gamma$ ranging from 0 to 1. To perform the numerical calculation, we discretize the spatial domain into $N=10$ equal intervals, each with a step size of *h*. The final solution is obtained at a specified final time *T* and stored in a matrix for each $\gamma$ value. Finally, to visualize the results, we plot the solution against the spatial variable *x*, with each line representing the solution for a different fractional order value.

All the tests are performed on a Windows 10 Pro operating system using Matlab version R2016b on a computer equipped with an Intel(R) Core(TM) i5-7200U CPU running at 2.5 GHz and with 8GB of RAM.

### Example I

16 $$\begin{aligned} \dfrac{\partial ^{\gamma (x,t)} \psi }{\partial t^{\gamma (x,t)}}=(1+x)\dfrac{\partial ^2\psi }{\partial x^2}+\psi , {\quad 0<\gamma (x,t)<1,} \end{aligned}$$

Subject to the conditions:

$$\begin{aligned} \psi (x,0)= & {} \psi (x)=x(1-x), \qquad 0< x<1, \\ \psi (0,t)= & {} 0,\quad \psi (1,t)=0 \qquad t\ge 0. \end{aligned}$$

**Solution:**

To obtain the discrete form of Eq. (16), the time fractional approximation (3) must be used for the time derivative and the central difference approximation for the space derivative.

$$\begin{aligned} \dfrac{{k^{-\gamma (x_i,t_{j+1})}}}{\Gamma (2-\gamma (x_i,t_{j+1}))}[(\psi _i^{j+1}-\psi _i^{j})+ \sum _{l=1}^{j}(\psi _l^{j+1-l}-\psi _l^{j-l})b_l^{i,{j+1}}]=(1+x_i)\dfrac{\psi _{i-1}^j-2\psi _i^j+\psi _{i+1}^j}{h^2}+\psi _i^j, \end{aligned}$$

Re-arranging the terms,

$$\begin{aligned} \psi _i^{j+1}&=r_i^{j+1}(1+x_i)\psi _{i-1}^j+(1-2r_i^{j+1}(1+x_i)+r_i^{j+1}h^2)\psi _i^j+r_i^{j+1}(1+x_i)\psi _{i+1}^j\nonumber \\ & \quad -\sum _{l=1}^{j}(\psi _l^{j+1-l}-\psi _l^{j-l})b_l^{i,{j+1}}, \end{aligned}$$

with

$$\begin{aligned} \psi _i^0&=x_i(1-x_i)\quad i=0,1,2,\ldots ,M.\\ \psi _0^j&=0=\psi _M^j\quad j=0,1,2,\ldots ,N. \end{aligned}$$

where $$r_i^{j+1}=\dfrac{k^{\gamma _i^{j+1}}\Gamma (2-\gamma _i^{j+1})}{h^2}.$$

**Figure 1:**
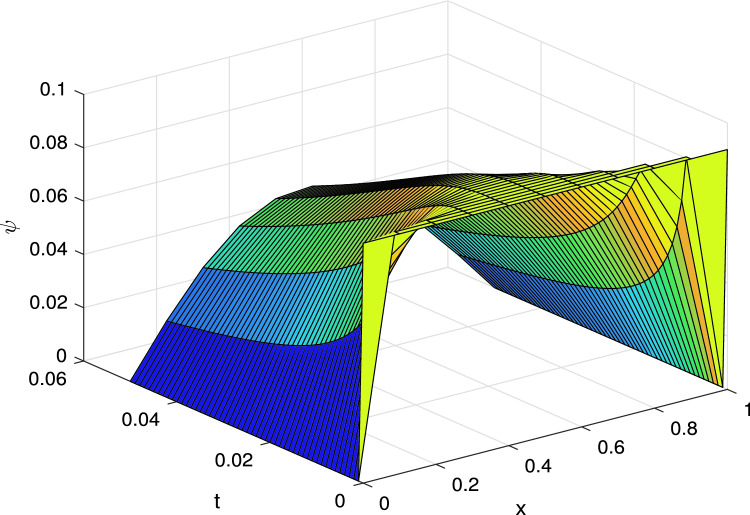
3D Solution curves for Example I.

**Figure 2:**
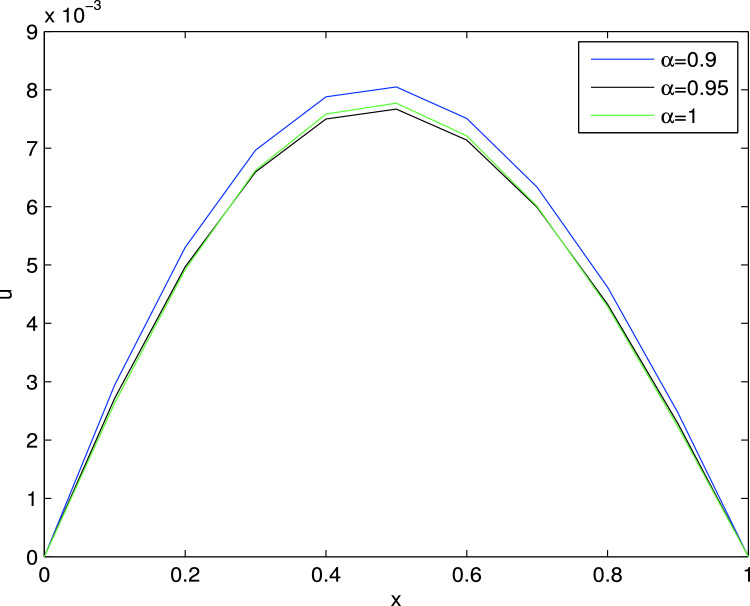
2D Solution curves for Example I.

The numerical solution for different values of $\gamma$ at the final time *T* is depicted in 3D Fig. 1 and also for 2D Fig. 2.

### Example II

17 $$\begin{aligned} \dfrac{\partial ^{\gamma (x,t)} \psi }{\partial t^{\gamma (x,t)}}=\dfrac{\partial ^2\psi }{\partial x^2 }-\dfrac{1}{x}\dfrac{\partial \psi }{\partial x} , {\quad 0<\gamma (x,t)<1,} \end{aligned}$$

Subject to the conditions:

$$\begin{aligned} \psi (x,0)= & {} \psi (x)=1-x^2, 0\le x\le 1.\\ \psi _x(0,t)= & {} 0,\quad \psi (1,t)=0, t>0. \end{aligned}$$

**Solution:**

Using time fractional approximation (3) the (17) can be written as

$$\begin{aligned} \dfrac{{k^{-\gamma (x_i,t_{j+1})}}}{\Gamma (2-\gamma (x_i,t_{j+1}))}[(\psi _i^{j+1}-\psi _i^{j})+ \sum _{l=1}^{j}(\psi _l^{j+1-l}-\psi _l^{j-l})b_l^{i,{j+1}}]&=\dfrac{\psi _{i-1}^j-2\psi _i^j+\psi _{i+1}^j}{h^2}-\dfrac{1}{x}\dfrac{\psi _{i+1}^j-u_{i-1}^j}{2h}, \end{aligned}$$

**Figure 3:**
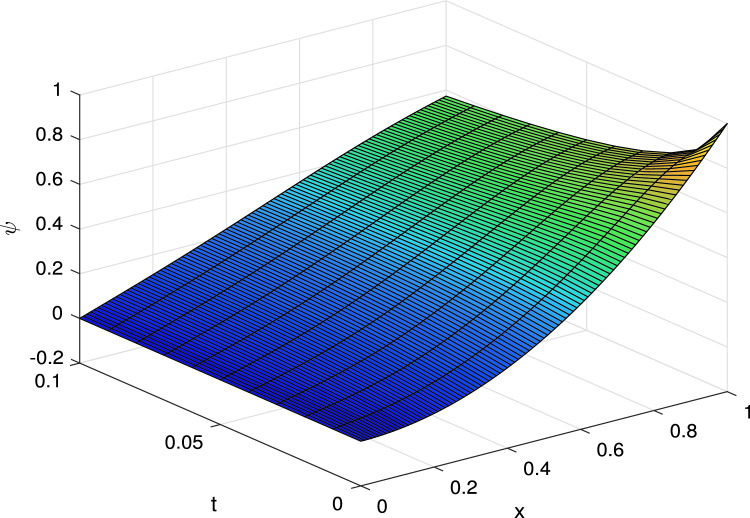
3D Solution curves for Example II.

**Figure 4:**
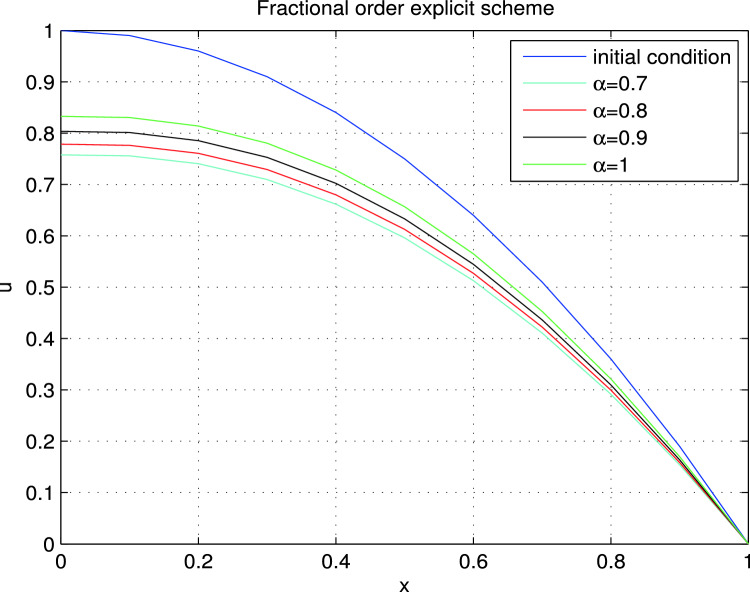
2D Solution curves for Example II.

Re-arranging the terms

$$\begin{aligned} \psi _i^{j+1}=\Big (r_i^{j+1}+\dfrac{r_i^{j+1}h}{2x_i}\Big )\psi _{i-1}^j+(1-2r_i^{j+1})\psi _i^j+\Big (r_i^{j+1}-\dfrac{r_i^{j+1}h}{2x_i}\Big )\psi _{i+1}^j-\sum _{l=1}^{j}(\psi _l^{j+1-l}-\psi _l^{j-l})b_l^{i,{j+1}}. \end{aligned}$$

with

$$\begin{aligned} \psi _i^0&=1-x_i^2\quad i=0,1,2,\ldots ,M,\\ (\psi _0^j)_x&=0,\quad \psi _M^j=0 \quad j=0,1,\ldots ,N, \end{aligned}$$

where $r_i^{j+1}=\dfrac{k^{\gamma _i^{j+1}}\Gamma (2-\gamma _i^{j+1})}{h^2}$. The numerical solution for different values of $\gamma$ at the final time *T* is shown in 3D Fig. 3 and 2D Fig. 4.

### Example III

18 $$\begin{aligned} \dfrac{\partial ^{\gamma (x,t)} \psi }{\partial t^{\gamma (x,t)}}=\dfrac{\partial }{\partial x}\Big ((1+x^2)\dfrac{\partial \psi }{\partial x}\Big ), {\quad 0<\gamma (x,t)<1,} \end{aligned}$$

Subject to the conditions:

$$\begin{aligned} \psi (x,0)= & {} 1000-|1000x|, \qquad 0\le x\le 1. \\ \psi _x(0,t)= & {} 0=\psi _x(1,t) \qquad 0\le t\ge 0.2. \end{aligned}$$

**Solution:**

After simplification, (18) becomes

$$\begin{aligned} \dfrac{\partial ^\gamma \psi }{\partial t^\gamma }=(1+x^2)\dfrac{\partial ^2\psi }{\partial ^2 x}+2x\dfrac{\partial \psi }{\partial x}, \end{aligned}$$

Applying the time fractional approximation (3) to the (18)

$$\begin{aligned} \dfrac{{k^{-\gamma (x_i,t_{j+1})}}}{\Gamma (2-\gamma (x_i,t_{j+1}))}[(\psi _i^{j+1}-\psi _i^{j})+ \sum _{l=1}^{j}(\psi _l^{j+1-l}-\psi _l^{j-l})b_l^{i,{j+1}}]&=(1+x^2)\dfrac{\psi _{i-1}^j-2\psi _i^j+\psi _{i+1}^j}{h^2}\\ & \quad +(2x)\dfrac{\psi _{i+1}^j-\psi _{i-1}^j}{2h}, \end{aligned}$$

Re-arranging the terms,

$$\begin{aligned} \psi _i^{j+1}&=((1+x_i^2)-hx_i)r_i^{j+1}\psi _{i-1}^j+(1-2r_i^{j+1}(1+x_i^2))\psi _i^j+((1+x_i^2)+hx_i)r_i^{j+1}\psi _{i+1}^j\\ & \quad -\sum _{l=1}^{j}(\psi _l^{j+1-l}-\psi _l^{j-l})b_l^{i,{j+1}} \end{aligned}$$

with

$$\begin{aligned} \psi _i^0&=1000-|1000x_i|\quad i=0,1,2,\ldots ,M,\\ \psi _0^j&=0=\psi _M^j \quad j=0,1,2,\ldots ,N, \end{aligned}$$

where

$$\begin{aligned} r_i^{j+1}=\dfrac{k^{\gamma _i^{j+1}}\Gamma (2-\gamma _i^{j+1})}{h^2}. \end{aligned}$$

In this research paper, we explore three examples of initial boundary value problems involving fractional partial differential equations. In Example I, we consider a problem governed by a time-fractional partial differential equation with a variable-order fractional derivative. The equation exhibits a linear and semi-linear variable-order time fractional characteristic. The Caputo derivative is employed to model physical memory and inheritance, and the problem is solved subject to specified initial and boundary conditions. Example II presents a different equation, also with a variable-order fractional derivative, but involving a different spatial derivative term. The equation includes a term that accounts for the singular behavior near the origin (at x=0) and requires additional boundary conditions at the right boundary (at x=1). Finally, in Example III, we explore a problem described by a time-fractional partial differential equation with a variable-order fractional derivative and a spatial derivative term. This example includes a non-homogeneous initial condition and satisfies zero-flux boundary conditions. By considering these three examples, we demonstrate the versatility and applicability of the explicit finite difference scheme in solving initial boundary value problems with various fractional characteristics, showcasing the effectiveness of the proposed approach.

## Conclusion and future work

This paper presents a novel explicit finite difference scheme specifically designed for solving initial boundary value problems with linear and semi-linear variable-order time fractional characteristics. The choice of employing the Caputo derivative in this scheme is motivated by its well-established significance in fractional calculus, enabling effective modeling of physical memory and inheritance. The thorough stability analysis using the Fourier method confirms the conditional stability of the proposed scheme. Numerical examples demonstrate the efficacy of the method, with graphical representations using MATLAB showcasing solution curves for different fractional orders. Moving forward, future research directions could include extending the scheme to more complex nonlinear problems, investigating adaptive mesh refinement techniques, exploring the application of the method to other scientific and engineering domains, and considering parallel computing techniques to enhance computational efficiency. This work contributes to the advancement of fractional partial differential equations solving methods and provides a foundation for further exploration and refinement of this approach.

## Data Availability

The datasets used and/or analyzed during the current study available from the corresponding author on reasonable request.
